# The Limits between Bipolar Disorder and Borderline Personality Disorder: A Review of the Evidence

**DOI:** 10.3390/diseases7030049

**Published:** 2019-07-05

**Authors:** Marsal Sanches

**Affiliations:** UT Health Department of Psychiatry & Behavioral Sciences, McGovern Medical School, Houston, TX 77021, USA; Marsal.Sanches@uth.tmc.edu or

**Keywords:** bipolar disorder, borderline personality disorder, diagnosis, neuroimaging

## Abstract

Bipolar disorder and borderline personality disorder are among the most frequently diagnosed psychiatric conditions. However, the nosological aspects and diagnostic boundaries of both conditions have historically been the object of considerable controversy. The present paper critically analyzes this debate, in light of available evidence. Clinical and neurobiological differences between bipolar disorder and borderline personality disorder are discussed, as well as the factors possibly involved in the overlap between both conditions and the potential implications of this.

## 1. Introduction

Bipolar disorder (BD) and borderline personality disorder (BPD) are among the most prevalent and frequently diagnosed psychiatric conditions. Both are associated with increased morbidity, elevated rates of suicide, and considerable functional impairment [1,2,3]. While the concept of BD has, over the past several years, been fully incorporated by culture [4], most of the general public is not yet completely familiar with the diagnosis of BPD, despite its popularity among psychiatrists and mental health providers.

Since the early 1980s, when BPD was included as a diagnostic category in DSM-III, considerable controversy has taken place with respect to its validity as a nosological construct. Initial discussions on the characterization of BPD emphasized the difficulties associated with its diagnosis and the distinction between the psychodynamic conceptualization of borderline organization and the DSM criteria for BPD [5,6,7]. On the other hand, the expansion of the so called “bipolar spectrum” concept has drawn attention to several phenomenological similarities between BPD and BD, to a lack of stability in the diagnosis of BPD over time, and to potential benefits in the treatment of BPD patients with mood stabilizers. Whereas several authors emphasized the difficulties commonly faced in the differential diagnosis of both conditions [8,9,10], a certain lack of consensus persists as to whether BD and BPD belong to the same spectrum.

The present paper addresses the limits between BD and BPD in light of available literature. It begins with a discussion of the core symptoms shared by both disorders, followed by a summary of the clinical evidence in favor for and against the existence of a “bipolar-borderline continuum”.

## 2. Why So Much Overlap between BD and BPD?

The DSM approach for mental disorders is, by definition, categorical. When it comes to the diagnostic criteria for BD and BPD, it is solely based on the description of symptoms. No well-established biological markers for either condition have been, so far, identified. Moreover, comorbidity between both conditions is not uncommon. Literature findings indicate the prevalence of BD type I among individuals with BPD ranges from 2.2 to 16.1%, while the rates of BD type II seem to vary from 4.8 to 18.7% [11]. In a recent study, a comprehensive literature review and meta-analysis of studies addressing the association between BD and BPD was performed [12]. Results confirmed the high prevalence of comorbidity between both conditions, with 21% of bipolar patients experiencing associated BPD. The findings also pointed out the possible involvement of age and gender effects in this association, with male gender and higher mean age predicting lower rates of comorbid BPD among BD patients. 

In contrast, the existence of less well-characterized categories under the BD umbrella, in combination with a marked overlap between some of the diagnostic criteria for BPD and BD [8], grants considerable latitude to clinicians when it comes to the distinction between both disorders, particularly in cases where full criteria for BD I or II are not met. For instance, the diagnosis of “unspecified BD”, according to the DSM-5, includes where “symptoms characteristic of a bipolar and related disorder” cause significant clinical distress or important impairment in different areas of functioning but “do not meet the full criteria for any of the disorders in the bipolar and related disorders diagnostic class” [13]. Despite its utility as a provisional diagnostic category, especially when limited collateral information is available, the category in question may leave clinicians open to several diagnostic bias, including countertransference issues, concerns about the stigma associated with one diagnosis or another, and personal misconceptions about the concept of a “soft bipolar” spectrum. This results in issues related to the possible underdiagnosis of BD, which has been repeatedly highlighted in medical literature over the last two decades, as well as concerns about the risk of over-diagnosing BD [14]. In a 2008 study with 700 outpatients, 56.6% of patients previously diagnosed with BD did not meet criteria for that condition, according to a structured interview [15]. Furthermore, among patients mistakenly diagnosed with BD, the prevalence of BPD was significantly higher than among those with no previous diagnosis of BD. 

## 3. A Continuum Between BD and BPD: Factors in Favor

Despite a proposed alternative model for the classification of personality disorders in the DSM 5, the criteria for BPD (as well as other personality disorders) were basically kept unchanged compared to the most recent DSM editions (i.e., DSM-IV and DSM-IV-R) [13]. The authors who support the inclusion of BPD in the spectrum of bipolar disorders argue that the DSM-based BPD construct lacks validity and steered the borderline concept away from its original psychodynamic characterization, corresponding to an amalgam of behavioral and cognitive symptoms, some of which overlap with features commonly found in patients with bipolar disorder [16]. These features include impulsivity, recurrent suicidal behavior, affective instability, and anger. A key distinction, according to the DSM, is that these symptoms seem to be chronic and pervasive in BPD, while they occur intermittently (i.e., during mood episodes) in BD. 

Nevertheless, in practical terms that distinction is far from clear, especially in the absence of a history of manic episodes. In BD type II, periods of hypomania may be difficult to distinguish from the patient’s baseline at times, and clinicians often have problems in identifying those periods retrospectively, unless reliable collateral information is available. Affective instability, as described in the DSM 5 criteria for BPD, can be characterized by quick shifts in mood, usually triggered by environmental factors which usually last a few hours and “rarely more than a few days”. This description is in marked contrast with more prolonged periods of mood polarization typically observed in true bipolar disorder (i.e., 14 days for depressive episodes, 4 days for hypomania, and at least 7 days for mania). DSM-5, however, admits the existence of several other forms of BD which do not meet the time criteria mentioned above. Moreover, some authors have proposed the existence of “ultra-rapid cycling” in BD, where quick shifts in mood could theoretically take place within hours. This makes it virtuously indistinguishable from the concept of affective instability described in BPD patients. 

Similarly, impulsivity is considered a core feature in BPD, but a large amount of evidence indicates that impulsivity is commonly found in BD patients and, rather than a symptom restricted to mood phases, seems to correspond to a trait-like feature that is present not only during acute episodes of mania or hypomania but also during remission [17]. Patients with BD have higher scores in the Barret Impulsivity Scale, a measure of trait impulsivity, than healthy controls. They also have high rates of comorbid substance use disorder and impulse control disorders, similar to patients with BPD [17].

Moreover, the overlap between BPD and BD becomes even more challenging when the concept of cyclothymic disorder is brought into the discussion. Classically considered a characterological condition, cyclothymic disorder was included as a subtype of BD in DSM-III and kept in its subsequent editions [18]. Despite the establishment of specific diagnostic criteria by the DSM, these are difficult to assess in clinical practice. The quick alternating of hypomanic and depressive symptoms in a short duration and the chronic nature of the condition indicate many similarities between this condition and BPD. According to Akiskal, borderline personality disorder could be regarded as the “dysphoric facet of cyclothymia” [16]. In this concept, cyclothymic disorder would be considered a temperament and BPD could emerge from cyclothymic disorder and be considered an unstable variety of the disorder characterized by dysphoric mood, explosivity, and anger episodes. This condition could be characterized under the denomination “BD-II ½” and would show strong similarity with atypical depression [19]. The author eventually hypothesizes that BPD, cyclothymic disorder, cyclothymia, and atypical depression would correspond to overlapping manifestations of a common underlying diathesis [16]. Of note, evidence does suggest that cyclothymic temperament traits are shared between bipolar and borderline patients [20,21].

Perhaps the most compelling evidence in favor of including BPD as part of the bipolar spectrum comes from longitudinal studies. These studies indicate that the diagnosis of BPD is not stable over time. Borderline patients are commonly found to no longer fulfil the diagnostic criteria for BPD at follow-up assessments, and symptom fluctuation also seems to be common [22]. These findings seem to bring into question the nature of BPD as a personality disorder itself, since DSM general criteria for personality disorders indicate that these traits are chronic, enduring, and pervasive. 

## 4. Neutral Findings or Evidence Opposing Borderline Personality Disorder as Part of the Bipolar Spectrum

### 4.1. Clinical Data Comparing BD and BPD

A limited number of studies have compared BD and BPD in terms of psychopathological features and performance in standardized instruments that aim for symptomatology quantification and characterization. In a study comparing patients diagnosed with BPD and BD utilizing the Mood Disorder Questionnaire (MDQ), three items (i.e., history of elevated mood, history of increased goal-directed activity, and episodic co-occurrence of manic symptoms) seemed to reliably differentiate both conditions, with a sensitivity of 88.7% and specificity of 81.4% [23]. These results were partially replicated by Balling et al. [24], who concluded that the discriminatory power of the symptom triad in question was higher than the one offered by the administration of the full MDQ. In another study [25], a factorial analysis including items of the MDQ and those of a screening instrument for BPD, the McLean Screening Instrument (MSI), administered to patients with BPD and BD indicated the existence of overlapping symptoms across both conditions (i.e., affective instability, impulsivity, irritability, flight of thoughts, and distractibility). Nevertheless, the remaining items of both scales seemed to be disorder-specific and, therefore, not shared across both conditions.

In a study by Bayes and Parker [9], the rates of patients assigned to a diagnosis of BD, BPD, or both according to clinical versus DSM-5 criteria were studied. The authors noticed that the rates of patients assigned to each group varied according to the criteria utilized. Of particular interest was the fact that the non-imposition of the DSM duration criteria for BP did not seem to impact the rate of BPD misdiagnosed as BD. 

Other authors have emphasized the phenomenological differences between the “mood swings” observed in BD and in BPD [26]. Despite their apparent similarity, key differences seem to be present between both conditions with regard to that construct. In BPD, brief mood swings that are triggered by external events and with a short duration do not seem to correspond to actual polarization in mood but to a different construct (i.e., affective instability). 

Lastly, a recent meta-analysis combined different studies analyzing temperament traits (measured through the Temperament Evaluation of Memphis, Pisa, Paris, and San Diego Auto-questionnaire or TEMPS) across different diagnostic categories, including mood disorder patients, their first-degree relatives, those with other psychiatric disorders (including BPD), and healthy controls [21]. The comparison between BD and BPD patients did not indicate significant differences with respect to cyclothymic temperament scores. On the other hand, several others temperament scores were markedly different between groups, including the hyperthymic temperament scores—which were higher among BD patients—and the depressive, irritable, and anxious temperament scores—which were found to be significantly lower in the BD group compared to the BPD groups.

### 4.2. Differences in Treatment Response

Finally, psychotherapeutic interventions seem to be the cornerstone of BPD management, and these patients usually show modest response to pharmacological treatment. Some reports suggest marked improvement in patients with BPD treated solely with pharmacological interventions [27], but results of randomized clinical trials are highly heterogeneous. For instance, in the only randomized clinical trial analyzing the effects of lithium on BPD symptoms, 17 borderline patients were treated with lithium, desipramine, or placebo in a cross-over design [28]. The authors identified no significant improvement in depressive symptoms associated with the use of lithium, although results suggested the positive impact of lithium treatment on anger and suicidality. The small sample size and the utilization of subjective measures (e.g., therapists’ perceptions on improvement) limit the generalization of the findings. In another double-blind, randomized, cross-over trial, four different medications (i.e., alprazolam, carbamazepine, trifluoperazine, and tranylcypromine) were compared to a placebo with regard to their effects on mood and behavioral deregulation [29]. Sixteen female patients not currently experiencing a major affective episode were included in the study. Results regarding the impact of interventions on mood were mixed, with objective improvements observed with tranylcypromine and carbamazepine, while subjective improvement (reported by patients) was observed only with tranylcypromine treatment. Carbamazepine produced important and significant decreases on the severity of episodes of behavioral dyscontrol, while alprazolam showed no impact on mood and an increase in the severity of behavioral deregulation. Nevertheless, another small randomized clinical trial on the impact of carbamazepine in the treatment of hospitalized BPD patients showed negative results [30]. 

Moreover, at least three randomized clinical trials analyzed the impact of valproic acid in the treatment of BPD patients. In the first one, 16 patients were randomized to receive either placebo or divalproex for a period of 10 weeks [31]. There were significant improvements in global symptom severity and functioning associated with divalproex treatment, although the small sample and high rates of drop-out were important limitations of this study. In another randomized, placebo-controlled trial, which included 30 female BPD patients with comorbid bipolar II disorder, treatment with divalproex sodium over a period of 6 months produced significantly positive improvements on interpersonal sensitivity, anger, and overall aggression [32]. Finally, in a third study, 15 patients initially treated with dialectical behavioral therapy for four weeks were randomized to receive either valproic acid or placebo [33]. No statistically significant differences were observed between groups. 

Lastly, two randomized clinical trials demonstrated the positive effects of lamotrigine in the treatment of BPD. In the first study, an eight-week treatment with lamotrigine was associated with reductions in anger [34]. In the second study, 28 BPD patients were randomized to either lamotrigine or placebo groups, with the former treatment showing significant effects in reducing affective instability and impulsivity [35]. 

Overall, the results of available clinical trials addressing the response of BPD patients to mood stabilizers indicate that these agents may produce positive effects on impulsivity but not on mood [26]. These findings support the characterization of BPD as a condition distinct from BD and not as part of the bipolar spectrum. 

### 4.3. Family Studies

If BD and BPD are part of the same spectrum, a certain degree of overlap with regard to genetic vulnerability to these conditions would be expected. That does not seem to be the case. While patterns of BPD familial aggregation have been identified in several studies, the inheritability of this condition seems to be considerably lower than that observed in BD patients [36]. Moreover, both conditions seem to show different patterns of familial aggregation, with evidence suggesting that BPD displays familial aggregation with impulse control disorders. Meanwhile, the aggregation with mood disorders seems to be ambiguous and is mostly accounted for major depressive disorder, not by BD [37]. In other words, the prevalence of BD among first degree relatives of BPD patients is similar to that found in the general population [38], which raises questions on the inclusion of both conditions in the same spectrum. 

### 4.4. Neuropsychological Studies

Neuropsychological impairment in bipolar patients has been extensively studied and supports the existence of fronto-limbic dysfunction in BD, even during euthymia. Compared to healthy controls, euthymic bipolar patients show impairments in executive function, attention/working memory, speed/reaction time, verbal memory, and visual memory [39]. Available evidence indicates the existence of strong correlations between the severity of cognitive deficits and the length of illness [40]. Moreover, it has been hypothesized that some of these deficits are also present in unaffected relatives of bipolar patients and may correspond to markers of vulnerability to BD [41].

On the other hand, while neuropsychological deficits in patients with BPD have been well described, the specificity of these findings may be questionable. In a meta-analysis of cognitive deficits in BPD [42], patients performed worse than controls in all domains analyzed, including attention, cognitive flexibility, learning and memory, planning, speeded processing, and visuospatial abilities. Of note, patients with BPD included in neuropsychological studies often present with comorbid psychiatric conditions, including major depressive disorder. In a cross-sectional study [43], borderline patients showed deficits in attention and immediate and delayed recall, with relatively preserved visuospatial and language functions compared with controls. Nevertheless, in this study, there were no significant differences between BPD and other personality disorders regarding neuropsychological deficits. 

### 4.5. Neuroimaging Findings

Neuroimaging findings in both BD and BPD point to the existence of abnormalities in the brain circuits involved in the modulation of emotions. In BD, structural and functional studies point to prefrontal dysfunction, with decreased modulation of subcortical structures [44]. Structural MRI studies in BD show large variability with respect to their findings, likely due to methodological issues related to acquisition parameters and characteristics of the samples (e.g., treatment status and mood state). Nevertheless, some findings seem to be more consistently replicated. These include larger lateral ventricles, smaller corpus callosa, decreased ventral prefrontal cortices, decreased anterior cingulate cortex volumes, and enlarged amygdala. The latter seems to be one of the most consistent findings in structural neuroimaging studies of BD patients [44]. 

Among BPD patients, on the other hand, the most common structural findings seem to be decreased amygdala volumes, decreased hippocampal volumes, and decreased orbitofrontal cortex volumes, as well as decreased anterior cingulate cortex volume [45]. With respect to functional studies, results from functional magnetic resonance imaging (fMRI) studies indicate elevated amygdala reactivity to negative or threatening stimuli in BPD patients [46], and that may be considered a neurophysiological correlate of affective instability. Decreased blood flow and metabolism in several prefrontal areas (including the dorsolateral prefrontal cortex and the anterior cingulate cortex) have been reported in BPD patients, and these findings may be partly responsible for the impulsivity and anger outbursts often observed in these patients. Such imaging findings, however, might be unspecific and are also present in BD patients [47].

In summary, despite the variability and lack of specificity of neuroimaging findings in BPD, available evidence suggests a slightly overlapping but an overall distinct pattern of findings between both conditions. Of note, there is scant evidence with respect to studies that specifically compared BD and BPD patients with regard to neuroimaging findings, and the high rate of comorbid major depressive disorder among BPD patients likely represents a huge confounding factor in the interpretation of the brain imaging findings in BPD patients. 

### 4.6. Other Biomarkers

The stress-diatheses model of BPD pathophysiology points to the role of environmental factors in the development of BPD and suggests that epigenetic factors are involved in the development of this condition. The findings of DNA methylation in certain genes has been considered an indicator of the involvement of epigenetic mechanisms in different conditions. In a 2011 study, BPD patients were found to have increased methylation in several candidate genes of other psychiatric disorders [48]. While the finding in question does not seem to be useful in the diagnostic delineation of BPD from other conditions, another study identified aberrant methylation of different CpG sites, including the APBA2 and APBA3 genes, among female BPD patients [49]. APBA2 and APBA3 are neuronal adaptor proteins, and aberrant methylation of their promoting genes have not been previously described in BD or other psychiatric conditions. 

Evidence on other candidate biomarkers in BPD patients does not seem to contribute to the distinction between BPD and BD. These biomarkers include the dexamethasone suppression test, the thyrotropin-releasing hormone test, and polysomnographic patterns [50,51]. 

## 5. Conclusions

Despite several clinical similarities between BD and BPD, available evidence suggests that both conditions are, from a nosological standpoint, distinct. Still, considerable overlap seems to exist between both conditions (Table 1). It is possible that some forms of bipolar disorder are virtually identical to BPD from a phenotypical standpoint, making both conditions difficult to distinguish at times, particularly given the absence of well-established biomarkers from both conditions. Considering the therapeutic and prognostic implications associated with misdiagnosing BPD as BD and vice-versa, studies aiming at improving the distinction between both conditions are of high interest.

## Figures and Tables

**Table 1:** Evidence supporting and opposing the existence of a continuum between BD and BPD.

Argument	Strength of Evidence	Comments
Psychopathological similarities	Medium	Some shared symptoms but several distinct features
High comorbidity between both conditions	High	High comorbidity rates not necessarily indicate continuum
Longitudinal instability of BPD criteria	High	Symptom fluctuation common in BPD
Shared temperament traits	Medium	Cyclothymic temperament common in both conditions. Otherwise, different patterns of temperament
Patterns of treatment response	Medium	Limited impact of pharmacological treatment on mood in BPD
Family studies	Medium	Different patterns of familial aggregation
Neuroimaging studies	Medium	Distinct findings in neuroimaging studies
Neuropsychological findings	Medium	Different neuropsychological findings
Other biomarkers	Low	Limited evidence
